# Supplementary material for: Splice-Junction-Based Mapping of Alternative Isoforms in the Human Proteome
Source: Cell Rep. Author manuscript; Available in PMC 2020 Jan 15. (PMC6961840; doi:10.1016/j.celrep.2019.11.026)
Supplement: 2 [file NIHMS1546469-supplement-2.zip › DF1/PXD006675/CardiacFibroblast/CardiacFibroblast_2_NEXN_NMEEEENETFGLSR.pdf]

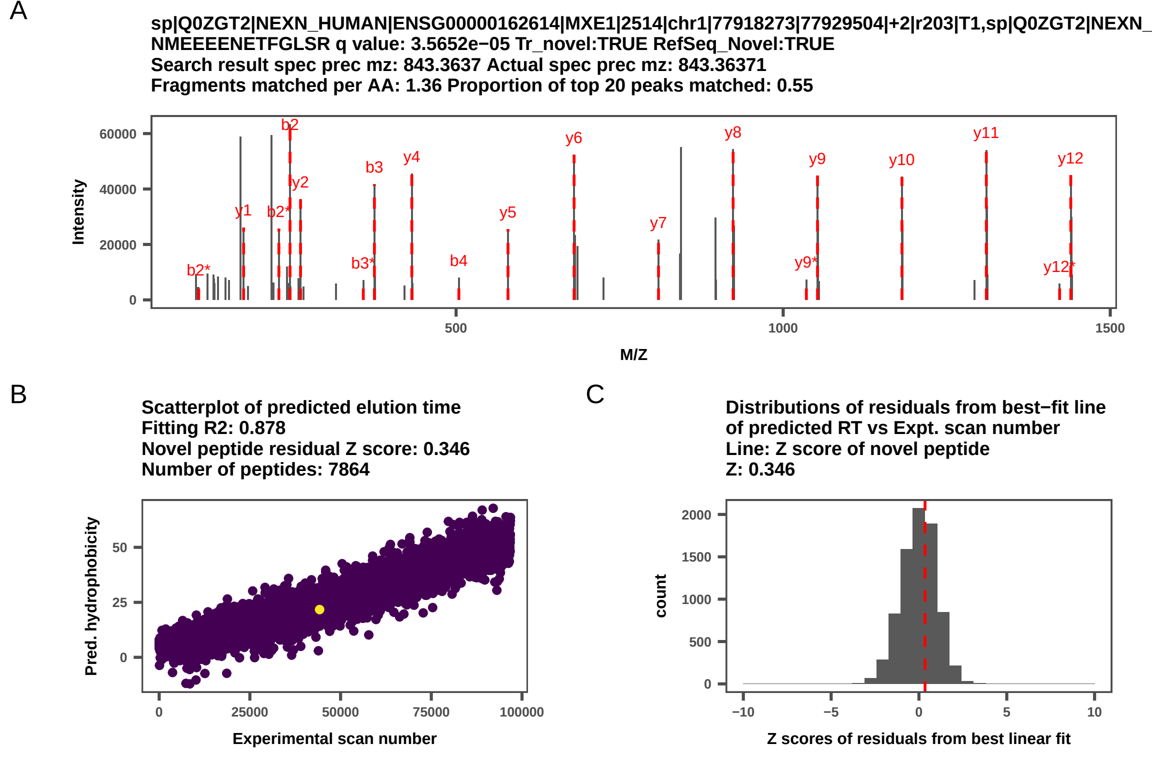
A
sp|Q0ZGT2|NEXN_HUMAN|ENSG00000162614|MXE1|2514|chr1|77918273|77929504|+2|r203|T1,sp|Q0ZGT2|NEXN_
NMEEEENETFGLSR q value: 3.5652e−05 Tr_novel:TRUE RefSeq_Novel:TRUE
Search result spec prec mz: 843.3637 Actual spec prec mz: 843.36371
Fragments matched per AA: 1.36 Proportion of top 20 peaks matched: 0.55
Intensity
60000
40000
20000
0
b2* y1 b2* b2 y2 b3* b3 y4 b4 y5 y6 y7 y8 y9* y9 y10 y11 y12* y12
500
1000
1500
M/Z
B
Scatterplot of predicted elution time
Fitting R2: 0.878
Novel peptide residual Z score: 0.346
Number of peptides: 7864
Pred. hydrophobicity
50
25
0
0
25000
50000
75000
100000
Experimental scan number
C
Distributions of residuals from best−fit line
of predicted RT vs Expt. scan number
Line: Z score of novel peptide
Z: 0.346
count
2000
1500
1000
500
0
−10
−5
0
5
10
Z scores of residuals from best linear fit
